# Usability, Acceptability, and Feasibility of a Personalized Adaptive Mirror Therapy for Upper-Limb Poststroke Rehabilitation Using Immersive Virtual Reality and Myoelectric Control: Single-Arm Pre-Post Study

**DOI:** 10.2196/81894

**Published:** 2026-05-04

**Authors:** Daniela De Bartolo, Paolo De Pasquale, Marta Russo, Denise Jennifer Berger, Antonella Maselli, Daniele Borzelli, Christian Nissler, Markus Nowak, Claudio Castellini, Giovanni Morone, Andrea d'Avella

**Affiliations:** 1 Clinical Laboratory of Experimental Neurorehabilitation IRCCS Fondazione Santa Lucia Roma Italy; 2 Faculty of Medicine and Psychology Department of Social and Developmental Psychology Sapienza University of Rome Roma, Lazio Italy; 3 IRCCS Centro Neurolesi Bonino-Pulejo Messina Italy; 4 Laboratory of Neuromotor Physiology IRCCS Fondazione Santa Lucia Rome Italy; 5 Institute of Cognitive Sciences and Technologies Consiglio Nazionale delle Ricerche (CNR) Rome Italy; 6 Center of Space Biomedicine Department of Systems Medicine University of Rome Tor Vergata Rome Italy; 7 Department of Biomedical Sciences, Dentistry, and Morpho-Functional Imaging University of Messina Messina Italy; 8 Institute of Robotics and Mechatronics German Aerospace Center (DLR) Weßling Germany; 9 Assistive Intelligent Robotics Lab Friedrich-Alexander-Universität Erlangen-Nürnberg (FAU) Erlangen Germany; 10 Department of Life, Health and Environmental Sciences, University of L'Aquila L'Aquila Italy; 11 Department of Biology University of Rome Tor Vergata Rome Italy

**Keywords:** assistive technology, digital health, mirror therapy, rehabilitation, stroke, users experience, virtual reality

## Abstract

**Background:**

Stroke remains a primary cause of long-term disability worldwide, with upper-limb deficits affecting up to 80% of survivors acutely and 40% chronically. These deficits lead to considerable effects on their independence and overall quality of life. Conventional rehabilitation therapies are most effective when initiated shortly after a stroke, yet many patients face barriers to ongoing therapy post discharge. Recent advancements in low-cost rehabilitation systems, particularly those using virtual reality (VR) technologies, offer promising alternatives for enhancing upper-limb recovery.

**Objective:**

Given the burden on health care systems and the limitations in access to high-intensity postdischarge rehabilitation, this study aimed to evaluate the feasibility, acceptability, and usability of an upper-limb adaptive mirror therapy using VR and myoelectric control for the rehabilitation of patients with chronic stroke developed through a user-centered design approach.

**Methods:**

In this study, a total of 12 community-dwelling survivors of chronic stroke (mean age 52.9, SD 16.0 years; 4 female) with moderate to severe upper-limb impairments were enrolled. Participants were stratified by age (young: 18-55 years; older: 56-80 years) and impairment level (Fugl-Meyer Assessment-Upper Extremity score: 18-36=severe; 37-54=moderate). Acceptability was assessed for each session by patient self-evaluation of satisfaction and motivation through a visual analog scale, while the therapist assessed the patient’s participation in therapy using the Pittsburgh Participation Rehabilitation Scale. Usability was measured with the User Satisfaction Evaluation Questionnaire scale and feasibility through the NASA (National Aeronautics and Space Administration) Task Load Index cognitive workload indices.

**Results:**

Patients reported a significant increase in satisfaction from the intermediate to the final assessment (T1: 72% vs T2: 85%; *P*=.01) and stable high motivation levels. Differences in participation and motivation were observed based on impairment levels, with no effect of age. Usability ratings remained high (>80%) throughout the intervention, with no significant differences between baseline and end line (*P*=.56). Cognitive workload assessments showed a significant reduction over time, in perceived cognitive (*P*=.04), performance (*P*=.007), and effort demands (*P*<.001). Impairment level significantly influenced perceived workload, with participants with more severe impairment reporting higher cognitive, physical, temporal, and effort demands (all *P*<.001), while age did not contribute to variability in acceptability, usability, or workload measures.

**Conclusions:**

VR therapy was found to be feasible, under adaptive task conditions, ensuring stable performance across patients. The protocol was usable and acceptable among patients with chronic stroke, especially those with moderate impairment, supporting its potential as a user-centered digital rehabilitation tool, warranting further investigation in controlled and home-based settings.

**Trial Registration:**

ClinicalTrials.gov NCT07103122; https://clinicaltrials.gov/study/NCT07103122

## Introduction

Stroke is one of the leading causes of disability worldwide. In Europe, 1.5 million people are diagnosed with stroke every year, costing European societies more than €60 billion [1]. As survival rates improve, life expectancy in general increases, therefore the burden of caring for stroke survivors is likely to increase [2,3]. About 80% of stroke survivors experience upper-limb deficits acutely, while 40% chronically [4,5], and of those with minimal movement on admission, only 11.6%-14.0% regain full function [6,7]. Motor impairments of the upper limb are particularly common and enduring for years after their onset. For this reason, the lack of upper-limb recovery results in significant loss of independence and a reduced quality of life, thus leading to reduced psychological well-being following stroke [8]. To be effective, conventional therapy must be delivered in the first months after the stroke onset with a high rate of intensity [9]. The literature shows that recovery of the upper limb and hand requires long periods of rehabilitation programs that need to be goal-oriented and involve cognitive stimulation [10].

However, due to several factors [11,12], the lack of therapy upon discharge home has resulted in challenges delivering the amount of rehabilitation necessary to optimize recovery [13]. For this reason, in recent years, there has been a focus on the development of low-cost rehabilitation systems that the patient can learn to use during hospitalization and can then use independently to continue the rehabilitation process at home [14]. In this setting, digital applications embedded in virtual reality (VR) systems have been suggested as rehabilitation tools to improve upper-limb recovery [15]. The advantage of these technologies is to provide a motivating treatment without requiring a long therapist contact time [16,17]. The enjoyable and challenging nature of such activities may help address issues of boredom, and, in addition, the ability to provide feedback may enhance motor learning [18], motivation, and exercise adherence [19], and therefore help provide the high-intensity, repetitive practice necessary to drive neuroplasticity-dependent functional recovery [20,21].

While VR rehabilitation systems may enhance the effectiveness of rehabilitation therapy, it remains to be established whether these new technologies can be suitable for every patient, namely, if they can be accepted and used as part of their recovery [22]. Addressing acceptability is not an easy task, as the user’s journey toward technological acceptance is complex and often nonlinear. A patient who decides to try a device may not use it in the long run; similarly, someone who has stopped using a system could go back to it later. Developers and researchers must address these issues in the design and evaluation process of new health care technologies, and across different stages of the users’ experience of the system.

A second aspect linked to acceptability is the feasibility of using a technological device for patients after stroke, according to motor severity. Several systematic reviews have already reported that both commercially available [23,24] and bespoke versions of VR systems [25] are feasible to use with positive effects on upper-limb recovery following stroke, for those with moderate and mild upper-limb deficits. Evidence in support of the use in patients with severe impairment is less convincing, with studies aiming to include patients with moderate to severe upper-limb impairments showing nonsignificant levels of improvement [26]. Moreover, many studies [27-29], including participants with moderate to severe upper-limb impairments, have used robotics or physical assistance from therapists in addition to VR, suggesting issues with the feasibility of the VR systems when used alone [27] or limited feasibility in the community of patients with stroke [26,30]. While critical to exercise adherence, few studies [21-23] have considered patient evaluation of VR devices, and when such evaluation has been performed, there has often been a lack of analytical rigor.

This study builds upon the technological framework and rehabilitation protocol that integrates VR with myoelectric control to establish a novel approach to mirror therapy (MT) for the rehabilitation of upper-limb neuromotor functions in patients after stroke [31]. The system architecture, control algorithms, and preliminary motor outcomes were previously reported in the framework of the user-centered design approach [32], primarily focusing on the technical validation of the system and its potential impact on motor performance. In contrast, this manuscript addresses a complementary and conceptually distinct objective, namely, the systematic evaluation of patients’ experience conceptualized as the feasibility, usability, and acceptability of the intervention.

In De Pasquale et al [31], a VR-based MT protocol was developed by adapting a virtual therapy arm system originally designed for the treatment of phantom limb pain in upper-limb amputees [33]. The system, based on a head-mounted VR display, motion trackers, and surface electromyography (sEMG) sensors, was repurposed for use in patients with stroke, with particular attention to the feasibility across a wide range of motor impairment levels. The protocol was designed so that the exercises are based on the patient’s level of motor proficiency and are thought to restore movements of daily activity. In fact, the virtual scene is the reproduction of different locations of a house in which the patient performs simple exercises that mimic the actions of daily life, such as reaching and grasping a virtual object through myoelectric control.

The integration of these characteristics into a rehabilitation protocol enables the effective use of the beneficial effects of MT, such as the motivational support provided by viewing the more affected limb moving [34], thus overcoming its weak aspects, such as its repetitive nature, limited therapeutic dosage, the necessity for specialized equipment, and professional supervision [35]. While conventional MT requires minimal physical equipment, it typically depends on continuous professional supervision to ensure correct execution and sustained engagement. The proposed VR-based system involves more specialized technological equipment but automates task delivery, feedback, and difficulty modulation, thereby reducing the need for constant supervision during therapy sessions once the system is set up. The addition of myoelectric interfaces to VR for MT rehabilitative protocols aids in interpreting the patient’s intentions by analyzing the residual electromyographic signals from the affected limb, so that patients can initiate voluntary movements by using their intact corticospinal pathways. This interaction enables the provision of feedback, such as realistic visual representations of a virtual limb, thereby creating a closed-loop system that fosters motor relearning and stimulates active engagement. As a result, this approach enhances motor coordination, boosts muscle strength, and alleviates spasticity [36,37].

Despite the established benefits of VR technology in enhancing motor functionality through immersive learning environments, evidence supporting the effectiveness of VR combined with MT protocols remains limited, primarily due to small sample sizes, suboptimal research designs, and low-intensity training [38]. In response to these challenges, we have developed a cutting-edge system designed to enhance the efficacy of traditional MT by harnessing the capabilities of VR with myoelectric control.

Accordingly, this study does not aim to assess clinical efficacy or motor recovery outcomes, but rather to investigate whether the proposed VR-based MT can be realistically adopted, sustained, and tolerated by patients with chronic stroke, including those with moderate to severe upper-limb impairment.

In this context, stratification by impairment severity was introduced to explore potential differences in feasibility, acceptability, and perceived workload across functional profiles, rather than to support definitive between-group comparisons. This exploratory perspective is particularly relevant in early-stage evaluations of complex rehabilitation or monitoring technologies [39], where understanding usability across levels of impairment represents a prerequisite for subsequent efficacy trials.

## Methods

### Ethical Considerations

This experimental study is part of the registered randomized clinical trial (ClinicalTrials.gov NCT07103122) and therefore uses an exploratory, single-arm pilot design focused on user experience and feasibility outcomes, without a control group. It was conducted in full compliance with the Declaration of Helsinki for research involving humans. The local ethical committee of the Fondazione Santa Lucia approved this research on December 2, 2019, (protocol CE/Prog. 790). Patients and caregivers were informed about the study procedures prior to enrollment into the study, and patients provided written informed consent.

Participant privacy and confidentiality were ensured in accordance with standard data protection procedures. All data were anonymized prior to analysis, stored on secure institutional servers, and access was restricted to authorized research personnel only. No financial compensation or reimbursement was provided to participants for their participation in the study.

### Participants

Based on our pilot study [31], we decided to enroll only patients with chronic stroke, who typically exhibit greater clinical stability, allowing for clearer assessment of treatment effects without the fluctuations seen in acute or subacute patients. Therefore, patients who had experienced a stroke more than a year before the first evaluation were enrolled for this study.

This study was designed as an exploratory pilot focusing on feasibility and user-experience outcomes rather than hypothesis testing. Therefore, the aim was to estimate usability/workload and feasibility parameters rather than to test efficacy; thus, a priori power analysis was not performed. Sample size was determined to provide adequate precision of key continuous user experience measures and to inform the design of a future adequately powered trial.

Criteria for enrollment were (1) upper-limb deficits with sufficient level of muscle power such that movement is possible with gravity eliminated (Medical Research Council [MRC] grade 2) or against gravity (MRC grade 3), as assessed using the MRC scale (grades ≥1) [40]; (2) scores ranging from 18/66 (27%) to 54/66 (80%) of upper-limb function, as assessed using the upper-limb extremity section of the Fugl-Meyer Assessment (FMA) scale [40]; (3) absence of severe linguistic impairments that may limit understanding of instructions; and (4) absence of cognitive impairments, as assessed with the Addenbrooke’s Cognitive Examination-Revised version for the Italian population [41], visual deficit, or other neurologic disease in comorbidity that may affect the patient’s ability to interact with the VR environment.

Clinical motor scales were collected at baseline to characterize the sample and stratify participants by impairment level; however, they were not analyzed as outcome measures in this manuscript. This choice reflects the exploratory nature of the study and the limited sample size, which suggested promising trends, although further investigation is needed to confirm rehabilitation efficacy.

Since age-related [42] and severity-related [7,26] differences were found in motor outcomes following VR upper-limb treatments, we created a blocked randomization list with 2 levels of stratification (age and severity), which guaranteed a balanced within-group split throughout the data collection process [43]. Therefore, participants aged between 18 and 55 years were considered young participants according to previous studies [44-46], while those aged between 56 and 80 years were considered older. The level of impairment was computed using 2 FMA-Upper Extremity–based cutoffs selected for functional relevance rather than normative severity thresholds. Patients scoring between 18/66 (27%) and 36/66 (54%) were classified as having severe impairment of the motor performance. This range reflects a markedly reduced voluntary motor control, typically associated with limited functional use of the upper limb.

Conversely, scores between 37 (56%) and 54 (80%) were classified as moderate impairment, as an indicative range of partial motor recovery and greater residual motor capacity, allowing for more consistent execution of goal-directed movements. The distinction between these 2 categories was chosen to ensure a clinically meaningful separation between patients with profoundly compromised motor function and those with a moderate, yet functionally relevant, level of recovery.

Given the limited sample size within each impairment subgroup, stratification was not intended to enable powered statistical comparisons, but rather to ensure representation of multiple functional levels of motor impairment, thus supporting an exploratory evaluation of feasibility-related outcomes.

We used the sealed envelope tool, whose implementation is based on Pocock [44], to create the randomization list of participants as a function of the stratification number of randomization blocks (age and level of impairment), which revealed the need to enroll at least 12 patients. This sample size was also used in previous work [16] about the effectiveness of the VR system on the rehabilitation of the upper limb in patients with stroke.

Although the rehabilitation protocol and technological platform are shared with the previous study [31], this analysis focuses on a different set of outcome measures, enrolled patients, and research questions. Specifically, the data reported here concern user experience, participation, usability, and perceived workload, which were not the primary outcomes of the earlier publication.

### Apparatus and Equipment

A detailed description of the equipment is described in previous publications [31,33]. Briefly, the system consists of an HTC Vive VR System, including a head-mounted display, 2 motion trackers, 2 controllers, 2 base stations, which emit infrared light and synchronization signals to enable accurate spatial tracking of the head-mounted display, controllers, and trackers, and 2 Myo armbands (Thalmic Labs). Each tracker of HTC and each Myo armband were positioned respectively on the hand dorsum and on the forearm of each participant. The tracker system provides the position and orientation of the patient’s hand. The Myo armband, consisting of 8 sEMG sensors, measures the forearm electric muscle activity of the user on the skin surface. The movement of the virtual hands is directly inferred from the kinematic data captured by the trackers, whereas the motion of the fingers is estimated based on forearm muscle activity. This estimation relies on a model trained using a machine learning approach consisting of an iterative version of random Fourier features ridge regression [47]. The acquired signals (tracker kinematic and sEMG) are transmitted wirelessly via Bluetooth. All processing, which includes signal acquisition, filtering, machine learning model training, and prediction, is carried out on an off-the-shelf laptop (Dell Alienware 15) with a dedicated GPU (NVIDIA GeForce GTX 1070).

### Software and the Virtual Environment

The rehabilitation software runs in Unity, a game engine platform commonly used for the creation of interactive 3D applications and VR environments. It consists of 3 different virtual environments in which a table in front of the patients matches the position and dimensions of a rehabilitation table. For each patient, an anonymous form is created and filled with the participant’s ID, date of birth, more affected side, and dominant hand. Before starting the training session, the program requires the acquisition of the positions of the table corners with 1 of the 2 controllers in use for the VR session for calibration, so that the virtual model of the table is spatially aligned with the corresponding physical table where the patient is performing the exercise. This was necessary to provide correct visuo-haptic feedback during the VR exposure.

### Procedure

After the clinical evaluation, each patient was enrolled for experimental training with the VR-MT. Before starting, a researcher explained the task to facilitate the patient’s interaction with the VR system. For all the patients, this was the first experience with a VR system in which myoelectric control must be exploited to achieve a task. The patient performed VR treatment while sitting on a chair with the possibility of resting their hands on a table. At the start of each session, the researcher carefully positioned the 2 Myo armbands over the brachioradialis muscles on each arm and attached the trackers to the back of the patient’s hands, aligning the z-axis between the index and middle fingers. As the system supports bimanual rehabilitation tasks by leveraging VR capabilities to provide mirrored assistance, assessment of proximal (limb movement) and distal control (hand gestures).

**Figure 1:**
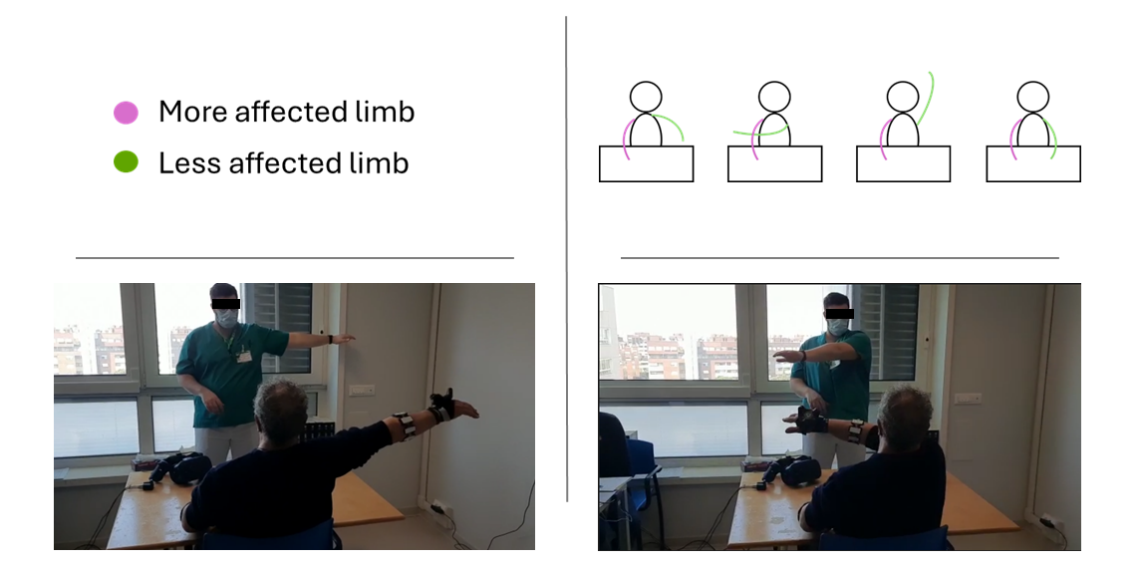
Example of a calibration procedure. In the right-upper part, a mannequin depicts the different steps involved in the calibration; in the lower part, 2 pictures show a patient performing the procedure before the beginning of the rehabilitation session.

Prior to each session, to personalize the training exercises by calibrating the reachable workspace the patient was asked to (1) assume a resting pose, with a comfortable position of the hand on the table, (2) extend the less impaired arm to the maximum range in the anteroposterior and mediolateral directions, as shown in Figure 1, and (3) extend the less affected limb in the same directions with the more affected limb, as much as they could. For each arm, the software evaluates the reachable area.

Although the calibration procedure visually emphasizes the movements of the less affected limb, the reachable space of the more affected limb is estimated based on the patient’s attempted movements with the more affected arm during the calibration phase. Specifically, patients were instructed to extend, first the less affected, and then the more affected limb in the anteroposterior, vertical, and mediolateral directions, with the latter moving as far as their residual motor capabilities allowed. The system records the maximum spatial extent achieved by the more affected limb during these attempts, which is then used as an estimate of its reachable workspace.

A secondary assessment was dedicated to calibrating the visualization of the opening and closing of a virtual hand (gesture) according to the electromyography signals recorded by the Myo armband. The patient is asked to (1) assume a resting pose with a comfortable position of the hand on the table and minimal muscle activity, (2) reproduce the gesture of holding a ball with the fingers extended, and (3) reproduce the gesture of holding the handles of a concertina with finger flexed.

After acquiring these 3 independent recordings, the researcher verifies the quality of gesture recognition and decides if the neural network needs more training or not. Otherwise, to improve the gesture reconstruction, it is possible to add new gesture repetitions, discard all the recordings of a gesture, or discard them all and redo the training gesture from scratch.

### Training Protocol

**Figure 2:**
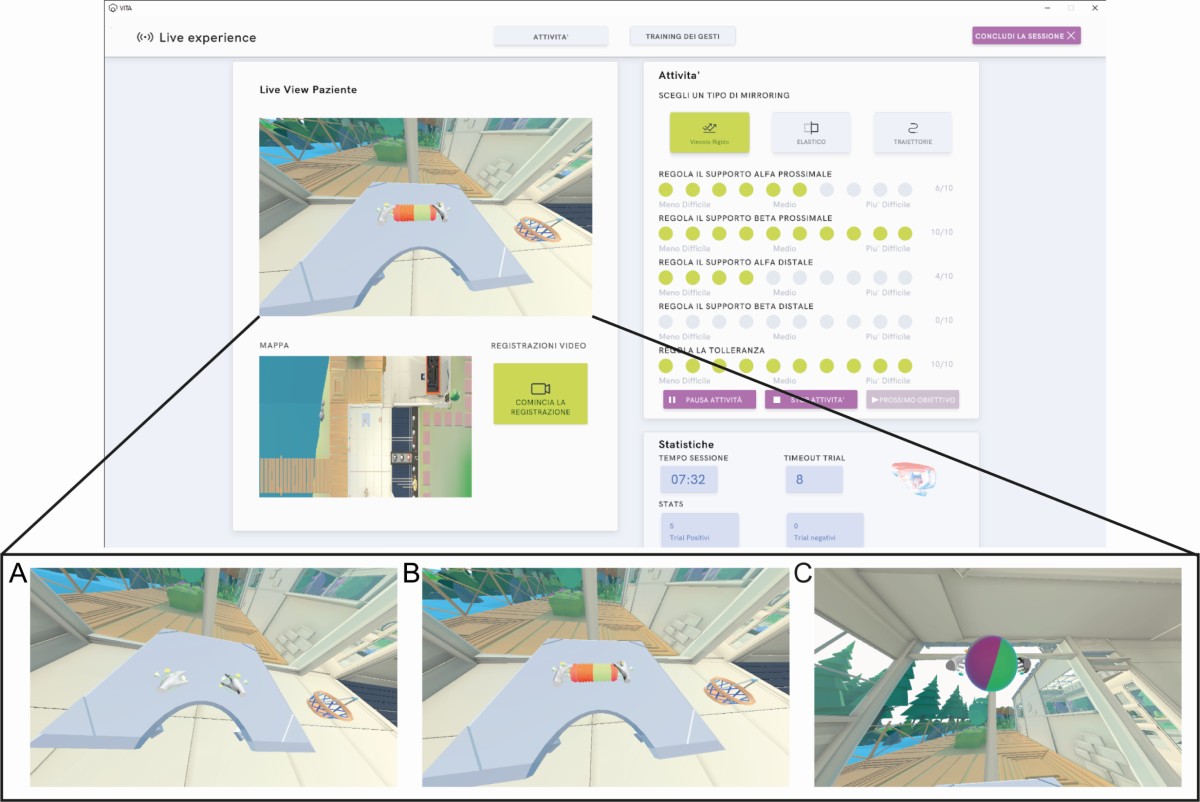
Overview of the Virtual Therapy Arm (VITA) interface and example of a session. The top panel shows the main user interface. On the left, the “Live View Patient” displays the real-time 3D visualization of the virtual environment from the participant’s perspective. On the right, the “Activity” panel summarizes session information, including task progression, performance indicators (green dots), and overall statistics (eg, total time and score). The bottom panels are representative of exercises available: (A) resting pose, (B) the fist gesture needed to grasp the concertina, and (C) the open hand gesture as to take a balloon.

The training comprised 24 sessions, each lasting about 20 minutes in a quiet room using a height-adjustable table and ergonomic seating to allow participants to perform reaching tasks in a physiologically appropriate posture, independently of body size. In the first session, the researcher encouraged the patient to visually explore the environment to facilitate familiarization with the VR scene, also avoiding possible distractions during the training. Then, the patient receives verbal instructions to perform the first tasks. The training involves 2 reaching virtual objects (a ball or a concertina) presented alternately to elicit different hand gestures. Specifically, interacting with the concertina requires performing a fist gesture (closing the fingers), while the ball requires a hand extension gesture (opening the fingers). Both the concertina and the ball feature 2 handles, positioned on opposite sides, to guide correct hand placement during the task, as depicted in Figure 2.

A reaching movement is considered successful when both virtual hands are positioned within 5 cm of the respective target handles. Real-time feedback on hand placement and gesture execution is provided within the VR environment. When both virtual hands are within the required tolerance, the handles change color from orange to green. Additionally, an on-screen gesture icon indicates the required hand posture and disappears once the correct gesture is performed. After both the position and gesture requirements are met, the target object gradually turns green, visually signaling the required holding time before task completion. Auditory feedback is also provided to enhance user awareness. A positive sound indicates successful task completion, while a negative sound is played if the trial exceeds the maximum allowed duration of 20 seconds. The position of each target varies in height and lateral deviation with respect to the initial resting position of the hands, which is recorded at the beginning of the session with the hands placed on the table. The maximum distance at which the target can appear is tailored to the maximum arm extension, while the minimum matches the resting position, both recorded by the patient prior to each session, for a total of 12 possible target positions. Targets were presented in a randomized order to prevent the repeated presentation of highly demanding trials, such as targets positioned near the limits of the participant’s reachable workspace or requiring more effortful hand gestures, which could otherwise induce premature fatigue. All training sessions were supervised by a physiotherapist, with specific attention to movement quality and prevention of compensatory strategies, such as excessive shoulder elevation or trunk displacement during reaching. Verbal feedback was provided to encourage controlled arm extension while maintaining proper posture. The patient is guided by the therapist to follow the instructions in the VR environment on how to place their hands inside the 2 spheres that appear next to the target object, with open hands in the case of the ball, with closed fists in the case of the accordion.

The first training session is dedicated to finding the right match among parameters according to the level of ability of the patient; therefore, a baseline level, which also serves as a familiarization phase to the experimental training. It consists of alternatively setting α and *β* parameters to 1 (independence by the less affected limb) and 0 (totally dependent). The baseline is intended to identify the optimal level of performance at the VR task for each patient; it must be difficult enough to create a challenging exercise but not excessively engaging, so that it can be sustainable for the duration of the training session without frustration. At the end of each block, consisting of 12 reaching movements, the therapist adjusts the proximal and distal agency and capability parameters based on the patient’s performance score. This score, calculated from the success rate within the block, guides the modulation of task difficulty for the subsequent block. When the patient achieves a success rate of >90%, corresponding to at least 11 successful trials, task difficulty is increased to promote greater motor engagement. If the success rate falls within 70%-90% (9-10 successful trials), task difficulty remains unchanged, ensuring the patient continues to perform within an optimal challenge level. Conversely, if the success rate is below 70% (≤8 successful trials), task difficulty is reduced to keep the task within the patient’s current capabilities and to prevent frustration or disengagement. This adaptive strategy was adopted to ensure that task performance remained within a sustainable range (ie, not falling below approximately 80%), thereby operationally defining feasibility as the ability to maintain engagement and performance over repeated sessions rather than complete task autonomy. Indeed, this framework is intended to sustain motivation and maximize motor improvement by continuously tailoring task difficulty to the patient’s performance, with the goal of maintaining a success rate within the target range of 70%-90%. The specific parameter to be adjusted, as well as the direction of adjustment, is determined by the therapist, who makes these decisions based on clinical expertise and real-time observation of the patient’s motor performance, fatigue, and overall condition. A complete description of how to set these 2 parameters to obtain virtual assistance is described in Multimedia Appendix 1.

### Acceptability, Usability, and Feasibility Evaluation

A total of 3 assessments with clinical scales and questionnaires were provided. A motor baseline at T0 (enrollment) was performed by a physiotherapist, which evaluated the patient’s muscle power through the MRC classification, and the residual motor proficiency with the FMA scale. Assessment was further performed at the intermediate evaluation (T1, after the completion of 12 sessions), and at the end of the therapy (T2, after the completion of 24 sessions). If the patients were discharged or expressed a willingness to interrupt the trial, the T2 evaluation was anticipated. Since acceptability reflects participants’ subjective evaluation of the intervention after experiencing it, a meaningful baseline assessment at T0 was not considered conceptually appropriate. At T0, participants had not yet interacted with the VR system and therefore could not provide an informed evaluation of satisfaction or motivation related to the intervention. For this reason, changes in acceptability were evaluated between the intermediate (T1) and final (T2) assessments, when participants had already gained direct experience with the therapy.

Acceptability is a multifaceted construct that reflects the extent to which people delivering or receiving a health care intervention consider it to be appropriate [48]. Here, we assessed this construct according to the emotional responses the patients exhibited during the intervention using 2 visual analog scales for motivation and satisfaction. As in a previous study [16], patients rated their level of motivation to attend the therapy and the satisfaction of doing the therapy for each session on a visual analog scale ranging from 0 to 10 (with a higher score indicating a higher level of motivation/satisfaction). In addition, the therapist/researcher assessed patient adherence to therapy, reporting on a Likert scale, ranging from 1 to 6 (where 6 stands for the highest level), the patient’s participation in the exercise through the Pittsburgh Participation to Rehabilitation Scale [49].

Usability, intended as the users’ potential to achieve their goals with effectiveness, efficiency, and satisfaction in a specified context of use of technology [50], was assessed using specific tools. Precisely, the User Satisfaction Evaluation Questionnaire (USEQ) [51] is a questionnaire properly designed to evaluate the satisfaction of the user in virtual rehabilitation systems. It is composed of 6 items assessing (1) the user’s experience with a VR system with respect to the enjoyment associated with the experience, (2) the success rate in using it, (3) the perceived control of the system, (4) the clarity of information provided by the system, (5) the level of comfort experienced, (6) and the evaluation of its impact for the patient’s rehabilitation. We performed this assessment at the first (T0) and last (T2) sessions of each patient journey, to evaluate their first impression of the VR system versus the final considerations acquired after multiple uses of the system.

Finally, in the framework of the person-based approach, we assessed the feasibility of our VR intervention as a workload [52] associated with the experimental VR therapy. For this assessment, we used the NASA (National Aeronautics and Space Administration) Task Load Index (NASA-TLX) [53] for the multidimensional subjective assessment rates of perceived workload associated with the task. Patients compiled this scale at the end of each training session, making an evaluation of the task according to mental, physical, temporal, effort, and frustration demands. Each domain is scored on a straight line that goes from 0 to 10 points (with 10 as the highest perceived workload), and the patients mark the point that corresponds to their evaluation.

### Statistical Analysis

The statistical analysis was performed using IBM SPSS statistical software (version 23), while customized algorithms were implemented in MATLAB (version 2020b; MathWorks) environments for data visualization. Data are reported in terms of means and SDs for continuous measures, as medians and IQRs for ordinal measures, and percentages for reporting variables expressed as frequency. The Kolmogorov-Smirnov test revealed a deviation from a normal distribution of all variables, thus nonparametric tests were adopted for comparing performance and ratings across the different times of evaluation (T0, T1, and T2). The Wilcoxon signed-rank test for related samples was used to assess the between-group analysis of differences in the self-assessed evaluation of patients after T1 and T2 sessions (after 12 and 24 treatment sessions, respectively). As a post hoc analysis, to assess differences within group (age and impairment), the Mann-Whitney *U* test was performed. The Spearman coefficient was further used to evaluate effect size in post hoc analysis to further test the contribution of impairment to the self-assessed cognitive workload domains. Analyses involving impairment level were conducted with an exploratory intent, aimed at identifying descriptive trends and potential patterns relevant to feasibility and user experience, rather than at testing formal hypotheses between subgroups.

Longitudinal usability outcomes were analyzed using linear mixed-effects models to account for repeated measurements and incomplete observations across sessions. Separate models were fitted for motivation, satisfaction, and participation after normalization to their respective theoretical maxima (motivation and satisfaction: 10; participation: 6), resulting in bounded outcomes in the range 0-1.

For each outcome *y_ij_*, measured for subject *i* at session *j*, the following model was specified:



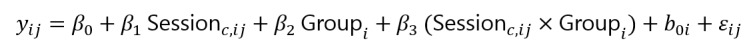



where Session*_c,ij_* denotes the session index centered around the sample mean, Group*_i_* represents the impairment group, *b*_0_*_i_* ~ *N* (0, *σ*^2^_b_) is a subject-specific random intercept, and *ε_ij_* ~ *N* (0, *σ*^2^) is the residual error term.

Models were estimated using maximum likelihood. Fixed effects were evaluated to characterize overall temporal trends, baseline group differences, and exploratory group-by-session interactions. Subject-specific random intercepts were included to account for within-subject correlation induced by repeated measures. Missing data were handled implicitly by the model under a missing-at-random assumption, without imputation. Given the limited sample size within impairment subgroups, all linear mixed-effects model analyses were considered exploratory and hypothesis-generating.

Only data from participants who completed a sufficient number of training sessions (≥10 sessions) were included in the usability analyses. This threshold was selected as a pragmatic criterion corresponding to approximately 3 weeks of repeated use, considered sufficient to move beyond initial familiarization effects and to obtain stable user experience measures, in line with usability research indicating that reliable usability estimates emerge after repeated interactions rather than during early exposure [54].

For all the tests, the significance was set at 0.05, except for post hoc analyses for which the α level of significance, according to Bonferroni correction, was divided by the number of possible comparisons (equal to 8), thus set at 0.006.

## Results

### Overview

**Figure 3:**
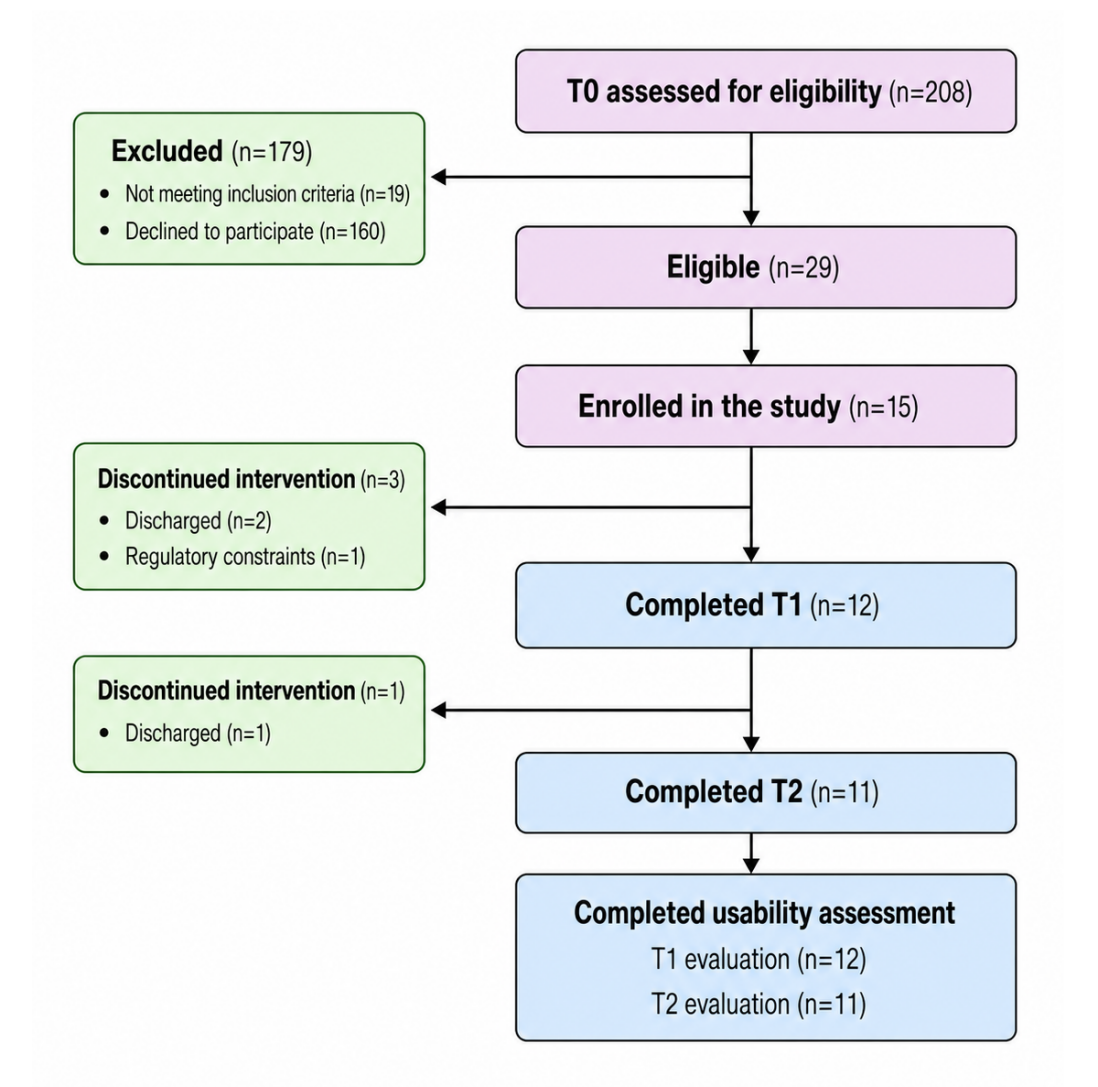
Participants flowchart. Research workflow at the 3 time points of assessment corresponding to the first evaluation (T0), after half of the treatment (T1), and the last (T2).

A total of 15 community-dwelling stroke survivors were initially recruited for the study. Of these, 12 community-dwelling stroke survivors (4/12, 33% females) aged between 27 and 72 (mean 52.9, SD 16.0) years completed a sufficient number of training sessions and were therefore included in the usability analyses. Three participants were excluded from the usability analyses due to insufficient exposure to the intervention, having completed fewer than 10 sessions. A detailed overview of participant flow is provided in the participant flow diagram (Figure 3).

Stroke chronicity was between 1 and 28 (median 2.9, IQR 1.8-8.4) years, and participants were classified as having moderate to severe motor impairment according to the FMA-Upper Extremity score. A total of 4/12 (33%) patients reported weakness following the stroke, on the right side of the body, and only 3/12 (25%) had a mild linguistic disorder (eg, anomia and dysarthria) that did not compromise either the patient’s ability to communicate or comprehend the verbal instructions given during the intervention sessions. Demographic and clinical information of all participants are presented in detail in Table 1.

**Table 1:** Demographic characteristics of patients with stroke. The data are presented according to age and motor classification. Motor impairment is quantified by the score obtained through the evaluation of the more affected limb with the Fugl-Meyer Assessment (FMA) scale, normalized to the total value of the upper extremity section and expressed as a percentage, as in a previous study [55]. Muscle power is reported as Medical Research Council (MRC) quality grade both for flexion (-f) and extension (-e) assessment of the more affected limb. The time since stroke (TSS) is reported in years; muscle power is the quality grade obtained with the MRC scale, assessed both for flexion and extension manipulative tasks. The side of weakness refers to the most affected side by stroke. In the “Training” column, the amount of training completion for each patient is reported.

Patient ID	Age			Motor impairment			Sex		TSS (years)		Muscle power			More affected limb		Linguistic deficit		Training (%)
Sub01	old	72	severe		45	Male		12		3		3	Left		No		100	
Sub02	old	72	severe		29	Female		2		2		2	Left		No		100	
Sub03	old	64	severe		41	Male		1		4		4	Left		No		96	
Sub04	old	70	moderate		62	Male		7		4		4	Right		No		83	
Sub05	old	64	moderate		82	Male		2		4		3	Right		Yes		100	
Sub06	old	58	moderate		73	Male		1		3		3	Left		No		92	
Sub07	young	48	severe		35	Male		28		2		2	Left		No		83	
Sub08	young	50	severe		52	Male		3		3		3	Left		No		100	
Sub09	young	27	severe		36	Female		4		3		3	Right		No		42	
Sub10	young	38	moderate		68	Female		25		4		4	Left		Yes		92	
Sub11	young	39	moderate		80	Male		2		4		4	Right		Yes		96	
Sub12	young	33	moderate		61	Female		3		4		4	Left		No		88	

All participants completed the whole experiment except for Sub09, who dropped out prior to the intermediate evaluation (T1); therefore, this participant was not included in the statistical analysis for T2 but was included for analysis at the other assessment points. Linear mixed-effects models showed consistently high levels of motivation, satisfaction, and participation across participants throughout the training period. Motivation exhibited a high baseline level and remained stable over sessions, with no significant effects of impairment group or session, and a nonsignificant trend toward a group-by-session interaction (group: *P*=.55; session: *P*=.31; group × session: *P*=.07). Satisfaction similarly showed high baseline values and remained stable over time, with no significant main effects or interactions (group: *P*=.72; session: *P*=.41; group × session: *P*=.86). In contrast, participation showed a significant positive effect of session, indicating a progressive increase over time, while no significant effects of impairment group or group-by-session interaction were observed (session: *P*=.01; group: *P*=.96; group × session: *P*=.37).

### Acceptability

**Figure 4:**
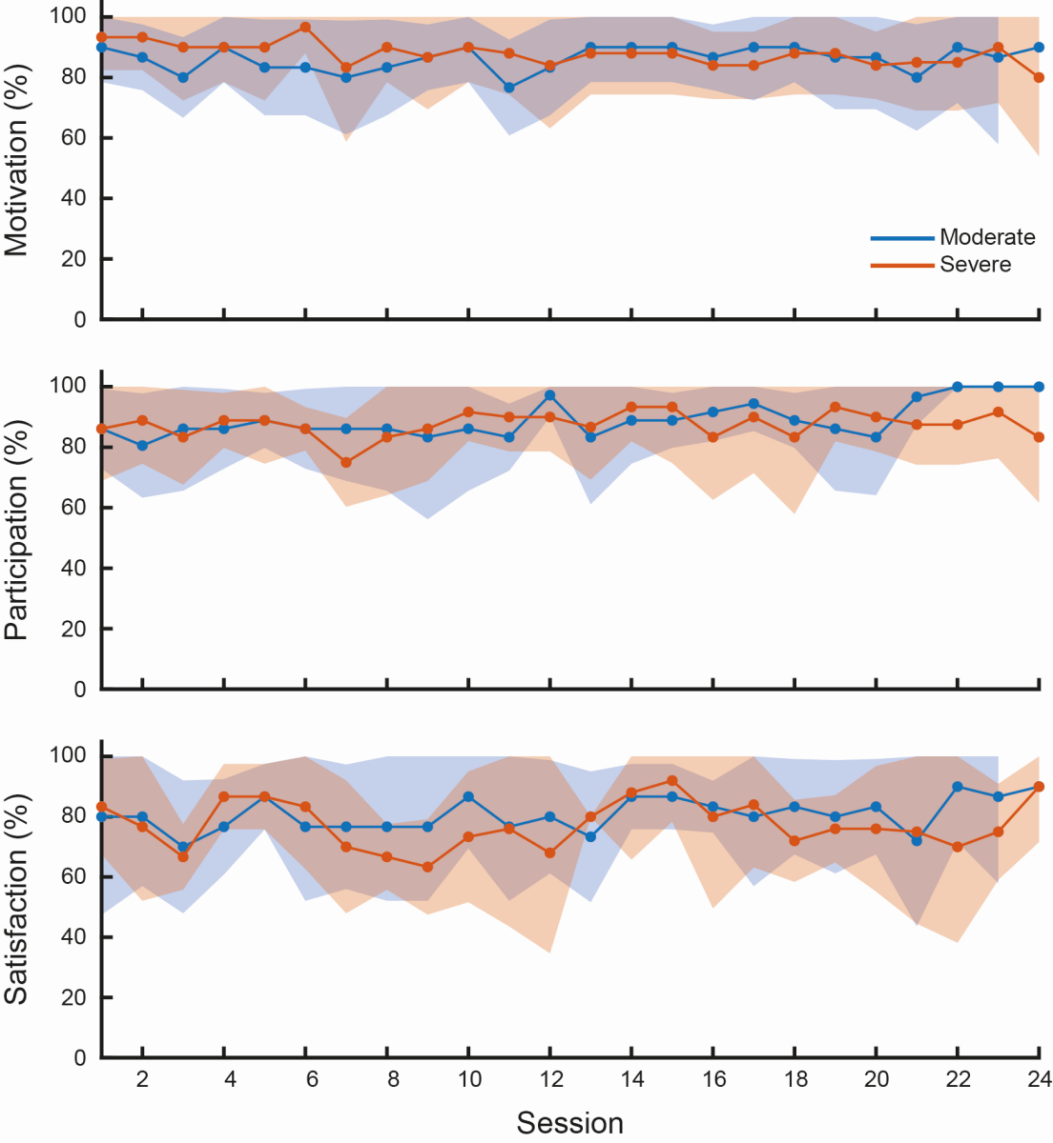
Session-wise trajectories of acceptability assessment. The longitudinal evolution of normalized scores of acceptability assessment, across the 24 training sessions, separately for patients with moderate and severe impairment. Scores were normalized to the theoretical maximum of each scale (motivation and satisfaction: 10; participation: 6) and are expressed as percentages. Solid lines represent the mean score at each session, while shaded areas indicate the 95% CIs. Missing sessions due to early discontinuation were handled by computing session-wise averages based on available observations only.

In line with the overall longitudinal trends, acceptability outcomes were further explored by comparing intermediate (T1) and final (T2) assessments to characterize clinically meaningful changes over time and across impairment levels. Comparisons of reported scores at T1 and T2 revealed a significant difference (*P*=.01, Wilcoxon signed-rank test) in the mean level of satisfaction declared by patients between the T1 (72%) and T2 (85%), as well as a significant difference (*P*=.01, Wilcoxon signed-rank test) in the mean level of patient participation assessed by the therapist at T1 (93%) and T2 (89%), but not in the level of motivation (*P*=.38, Wilcoxon signed-rank test) which for moderate patients always remained very high as shown in Figure 4. Within-group analyses did not highlight differences explained by age for any of the parameters investigated. Indeed, adherence to therapy, assessed as the level of motivation expressed by patients and participation assessed by the researcher/therapist, was different for patients according to the level of impairment (motivation: *P*=.02; *r*=0.142; participation: *P*=.045, *r*=–0.124; Wilcoxon signed-rank test and Spearman coefficient of correlation, respectively).

### Usability

**Figure 5:**
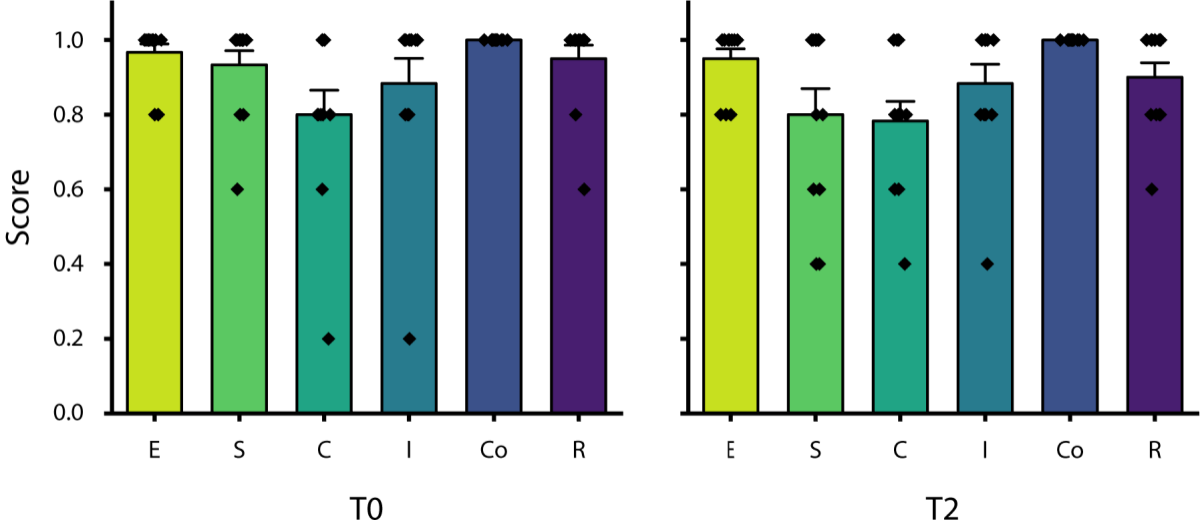
Usability assessment. The score of the User Satisfaction Evaluation Questionnaire obtained at baseline (T0, left panel) and end line (T2, right panel). Domains of usability were assessed as enjoyment (E), success (S), control (C), information (I), comfort (Co), and helpful for rehabilitation (R). Each bar is the mean score obtained for each domain of the User Satisfaction Evaluation Questionnaire, with error bars computed based on SD. Dots are representative of users’ evaluations for each domain.

We asked patients to evaluate the device used for VR motor training according to the item of the USEQ scale reported in Figure 5. The patient performed this evaluation after the completion of the first training session (baseline, T0) and after the last one (end line, T2). All domains assessed with this scale scored at least more than 80% up to 95% in the first session, with small variation in the last one. Therefore, we did not find any difference between the two assessments (*P*=.56, Wilcoxon signed-rank test).

Importantly, the increase in satisfaction over time reflects patients’ growing engagement and acceptance of the therapy, whereas the stable usability scores indicate that the system did not require a prolonged learning phase and was perceived as usable from the outset.

### Feasibility

**Figure 6:**
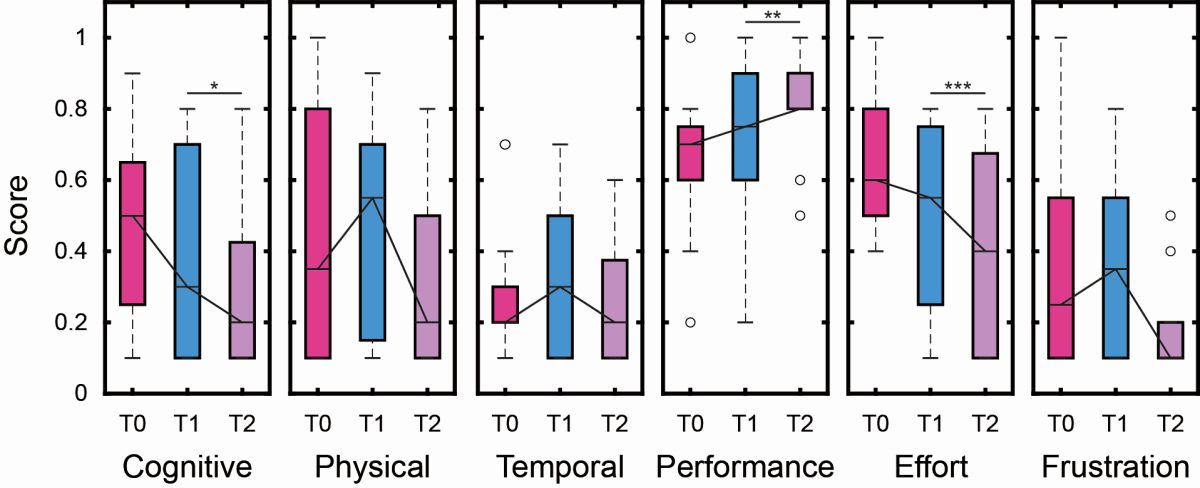
NASA Cognitive Workload Index Assessment. Each panel shows a domain of workload assessed through the rehabilitation protocol, with the three boxplots reporting the distributions at each time of the assessment (T0, T1, and T2). Median values have been linked with a solid line to highlight the trend of each domain across the three evaluations. The whisker edges represent the first and third quartiles of each distribution, while empty dots represent the outlier values. Statistically significant differences between the intermediate (T1) and the final assessment (T2) are indicated by brackets above the corresponding boxplots. Significant improvements were observed in the cognitive, performance, and effort domains (Wilcoxon signed-rank test, *P<.05, **P<.01, ***P<.001).

Patients’ evaluation of cognitive load associated with the VR-MT training was assessed through the NASA-TLX cognitive index as shown in Figure 6, in which differences between the intermediate (T1) and the final evaluation (T2) were found for cognitive (*P*=.04, Wilcoxon signed-rank test), performance (*P*=.007, Wilcoxon signed-rank test), and effort domains (*P*<.001, Wilcoxon signed-rank test). As for acceptability measures, within-group analyses showed that age did not contribute to the variance of NASA domains in the longitudinal evaluation of the treatment.

**Figure 7:**
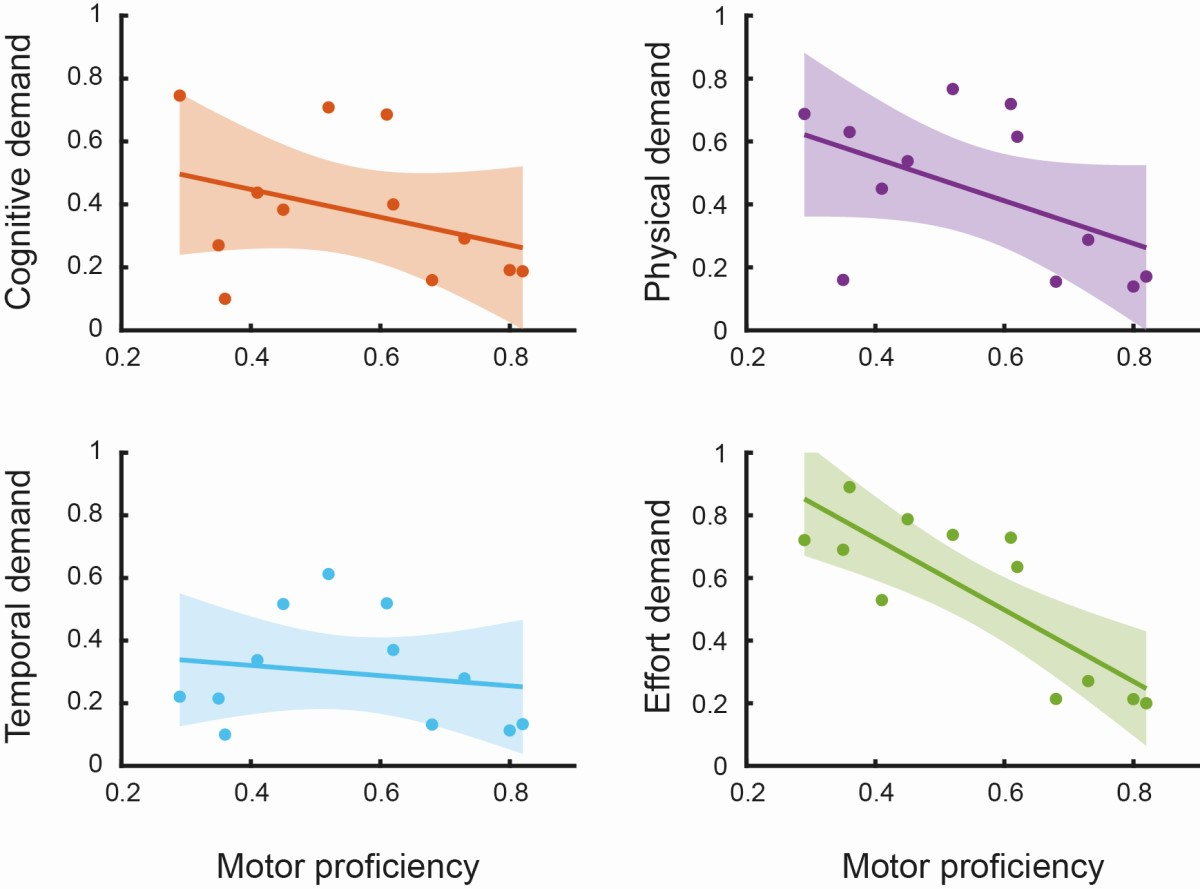
Post hoc comparison of NASA Task Load Index scores. The figure shows, in clockwise order from the first panel on the top left, the scatter plot between motor proficiency and the NASA cognitive, physical, temporal, and effort demands. Each point represents the patient’s overall assessment of the training according to the considered demand; the solid line is the fitted linear model with the variable motor proficiency as a predictor of the scores for the individual NASA demands. The shaded areas represent the CIs of the model set at 95%.

On the contrary, post hoc analyses with Mann-Whitney *U* test for independent samples and Spearman correlation coefficient as depicted in Figure 7, clearly show that cognitive workload is perceived differently according to the level of impairment for cognitive (*P*<.001; *r*=–0.462), physical (*P*<.001; *r*=–0.422), temporal (*P*<.001; *r*=–0.408) and effort domains (*P*<.001; *r*=–0.317).

Notably, differences between impairment subgroups were more evident in measures related to perceived workload and participation, particularly in patients with severe impairment. While these observations cannot support inferential conclusions, they are consistent with clinical expectations and reinforce the relevance of impairment severity as a key factor in feasibility evaluations.

## Discussion

The integration of immersive VR-MT in neurorehabilitation has shown promising results for enhancing motor recovery in patients with upper-limb impairments following neurological injuries. However, the effectiveness of training in immersive VR versus conventional therapy is still confined to expert opinion due to heterogeneity of study design, small sample sizes, and differences in the level of impairment of patients enrolled [56,57]. Recent studies suggest that rehabilitation delivered through VR systems may facilitate upper-limb motor and functional recovery and improve health-related quality of life after stroke, while also showing promising results in terms of feasibility and acceptability in populations with chronic stroke [16,18,21,25,38,58]. More precisely, VR protocols designed to embed MT principles have also reported good results in eliciting psychophysical effects such as tingling or paresthesia in the affected limb during the VR experience [58].

The aim of this study was to provide meaningful insights into the feasibility, usability, and acceptability of the adaptive MT using VR and myoelectric control for the rehabilitation of stroke survivors with moderate to severe upper-limb impairment. From a rehabilitation perspective, patients with severe upper-limb impairment represent the most critical test case for the feasibility of VR-based interventions. The exploratory patterns observed in this subgroup, particularly in terms of workload and participation, support the feasibility of the proposed system even under more challenging functional conditions, which is a key requirement for real-world adoption.

Our findings indicated that patients were satisfied with the training they underwent, maintaining a high level of motivation and participation in therapy, although this varied greatly across sessions and with specifics depending on the level of motor impairment. The system’s usability assessment, similarly, maintained a very high rating since the first session. Conversely, the workload assessment associated with the task revealed a different perception in terms of cognitive, physical, time demand, and effort for severely impaired patients, who scored higher than moderately impaired patients.

In the context of increasing stroke incidence and improving survival rates, the need for sustainable and accessible rehabilitation options is fundamental [59]. Considering the demographic transition toward an aging population and an increasing prevalence of chronic conditions, including stroke, the inclusion of innovative technological solutions in clinical and home-based rehabilitation pathways is becoming more urgent and frequent. Furthermore, the long-lasting sequelae following stroke demand a sustainable and motivating therapeutic approach that addresses the patient’s therapeutic needs and, at the same time, keeps a stable motivation to continue the rehabilitation program.

Although this study was conducted in a supervised clinical setting, the findings provide relevant insights into the potential feasibility of future home-based use. The absence of physical assistance during task execution, together with stable usability and sustained participation, suggests that selected patients, particularly those with moderate impairment, may be considered for future semisupervised or remotely monitored applications. The system was designed to automate task delivery, feedback, and difficulty adaptation, which are key prerequisites for reducing reliance on continuous therapist supervision. However, these results should not be interpreted as evidence of feasibility in a fully autonomous home setting. Indeed, future studies will be needed to support fully autonomous home use and indicate the need for additional safety mechanisms and structured therapist oversight before implementation in unsupervised settings.

Our study aligns with and extends current literature on the need for technological tools integration into poststroke rehabilitation settings [60,61], particularly by quantifying user experience in terms of usability of the VR motor program for upper-limb recovery, as well as of motivation and satisfaction.

Our data showed encouraging outcomes regarding user satisfaction and therapy participation, with significant improvement in both parameters between the intermediate and final evaluations (*P*=.01 for both). This result underscores the potential of VR systems not only as therapeutic tools but also as motivational enhancers throughout the recovery process. While motivation levels remained high across the study period regardless of time (*P*=.38), the fact that satisfaction increased significantly indicates that continued exposure and growing familiarity with the system positively influenced the patients’ overall rehabilitation experience. These findings support earlier observations [62] that the engaging, interactive nature of VR environments can sustain user interest and potentially enhance neuroplastic changes through intensive, repetitive activity.

Crucially, our data also emphasizes that VR-based rehabilitation therapies must be adaptable to patients’ functional capabilities. In fact, in our study, we included patients with different levels of motor proficiency to assess how this aspect could significantly influence adherence to therapy and the associated cognitive workload. The observed effects of impairment level on therapy participation (*P*=.045; *r*=–0.124) and motivation (*P*=.02; *r*=–0.142) point to a nuanced relationship between physical function and engagement, echoing concerns raised by Laver et al [26] regarding the limited benefits of VR interventions for individuals with more severe motor limitations unless additional assistive elements (eg, robotics or therapist involvement) are introduced. Our results are consistent with those of Chen et al [25] and Kim et al [30], who found that feasibility and therapeutic outcomes are significantly modulated by the severity of disability, and that user-centered design is imperative to address this diversity.

In this study, task difficulty was deliberately personalized through adaptive modulation of assistance and capability parameters, resulting in different absolute task demands across participants.

Importantly, as in similar feasibility research [52,63,64], NASA-TLX scores were interpreted as subjective indicators of perceived workload and feasibility rather than as proxies of motor improvement. While reductions in workload over time may partially reflect a system-related learning curve, they occurred within an adaptively regulated training protocol in which task difficulty was progressively adjusted. Therefore, longitudinal changes in NASA-TLX likely reflect the participants’ evolving balance between increasing task demands and improved familiarity and efficiency in interacting with the system, rather than a reduction in task challenge or direct motor recovery.

Consequently, NASA-TLX scores should not be interpreted as a direct reflection of task difficulty per se, but rather as an index of the individual’s perceived effort relative to their own functional capacity, cognitive resources, and motor impairment.

From this perspective, variability in NASA-TLX scores across participants is expected and informative, as it captures differences in perceived cognitive and physical demand associated with performing a personalized task. Our results show that impairment severity has a significant impact on perceived workload as assessed through the NASA-TLX scale. Differences in cognitive, physical, temporal, and effort demands were all significantly influenced by impairment level, particularly in post hoc comparisons (eg, cognitive: *P*<.001; *r*=–0.462), where higher motor proficiency is associated with a lower level of perceived workload. The observed association between impairment level and perceived workload suggests that, even under adaptive conditions, participants with more severe impairment experienced higher cognitive and physical demands. This finding provides meaningful insight into feasibility, highlighting the need to carefully balance task challenges and user burden when designing adaptive VR-based rehabilitation interventions. While the therapy was generally well-received, its design and implementation must remain sensitive to the cognitive and physical demands placed on users with greater motor impairment. Systems that fail to tailor difficulty and feedback mechanisms to the user’s ability level risk inducing cognitive overload, decreased motivation, and ultimately discontinuation of use. The inverse relationship we found between impairment severity and perceived cognitive load, frustration, and effort suggests that the simplified interfaces and personalized training pathways delivered through our approach played a critical role in fostering sustained engagement in this group of patients.

Despite the additional challenge faced by patients with severe impairment, usability scores across all domains remained above 80%, both at the beginning and end of treatment. Patients gave high scores to the system in all domains assessed by the USEQ scale, with most of them maintaining 80%-95% approval from baseline to the end of the intervention. The stability of these results indicates that the system is not only initially user-friendly but remains so throughout the rehabilitation course. These findings are in line with a review of literature [65] addressing the problem of whether patients with varying degrees of motor impairment benefited from technology-assisted interventions, particularly when those interventions were intuitive and adjustable according to patient capabilities.

Although many interventions are designed with a primary focus on functional gains, fewer consider how the patient perceives the treatment, whether they enjoy it, feel comfortable using it, and believe it contributes to their recovery. This focus on user satisfaction and emotional response is increasingly recognized in the literature as essential for optimizing outcomes in digital health interventions [22,66]. As demonstrated in our findings, user satisfaction and motivation increased over the course of treatment, even if no significant changes in the objective interface or content of the application were made. This suggests that familiarity with the tool, confidence gained through usage, and a sense of mastery may contribute to increased acceptability over time. This finding aligns with more recent evidence suggesting that immersive VR applications can effectively facilitate adherence in neurological rehabilitation by providing a sense of agency (ie, perceived control over the virtual limb) and progression in the rehabilitation path [67,68].

We did not find any significant age-related differences in acceptability, usability, or cognitive load perception, suggesting that older adults can also effectively engage with and benefit from VR-based rehabilitation when the system is designed appropriately. These findings contribute to a growing body of work [69,70] that supports the notion that age itself is not a barrier to the acceptance of digital health technologies; rather, usability and personalization are the defining factors for acceptance.

Importantly, our study showed that the level of participation as assessed by the therapist remained high across both intermediate and final evaluations, with only a modest decrease. This suggests that while the initial novelty of the technology may taper over time, the structured and tailored nature of the VR training maintains its overall therapeutic value. Other studies have similarly shown that tailored rehabilitation technologies designed according to the principles of user-centered design yield higher satisfaction and usability scores than standardized or generic programs [16,25,31].

Finally, the broader implications of our study support recent discussions advocating for the inclusion of technology-based rehabilitation in standard care protocols, particularly given the limitations of conventional rehabilitation services in terms of accessibility, cost, and therapist availability post discharge [71,72].

This study has several limitations that should be acknowledged. First, postintervention clinical motor outcomes were not included, which limits the ability to draw conclusions regarding rehabilitation efficacy. This choice was intentional and reflects the role of this study within a broader user-centered design process guiding the development of the intervention. In this framework, the primary objective was to assess feasibility, usability, and acceptability from the user’s perspective, as foundational steps in the iterative refinement of a technologically complex therapeutic system, rather than to quantify motor recovery outcomes, which fall within subsequent phases of clinical evaluation.

Second, given the relatively small sample size and the single-arm exploratory design limitation, the findings should be interpreted with appropriate caution. Nevertheless, this study was not intended to provide definitive evidence of effectiveness, but to generate preliminary, hypothesis-generating insights into user experience across different levels of motor impairment. In this context, the inclusion of participants with heterogeneous motor severity represents a strength, as it allowed the identification of differential patterns of participation, motivation, and perceived workload that may inform future personalization strategies. Accordingly, subgroup-related findings should be interpreted as exploratory and hypothesis-generating, and not as evidence of differential effectiveness between impairment levels.

Indeed, therapist assistance time, learning curves, and levels of independence during training were not explicitly quantified. Feasibility was intentionally operationalized within a patient-centered framework focused on sustained engagement, stable task performance under adaptive conditions, and acceptable perceived workload. Accordingly, the absence of age-related differences in feasibility outcomes should be interpreted within these boundaries. Future studies will be needed to integrate clinical motor outcomes and to quantify therapist involvement, autonomy progression, and learning trajectories in order to determine the effectiveness and real-world clinical impact of the intervention.

Finally, task difficulty and virtual assistance were dynamically adjusted to accommodate fatigue or reduced motor control, thereby limiting excessive physical strain. Although no adverse events were observed, safety outcomes were not systematically quantified. Future developments, particularly aimed at unsupervised or home-based use, should integrate automated detection of compensatory or unsafe movements, for example, by monitoring head or trunk position, to enhance safety and support independent training. Although we are still far from thinking that a VR-based therapy can completely replace the therapy performed by a physiotherapist, some important goals have been achieved. Unlike previous studies [26-29], the proposed system was designed to accommodate patients with severe motor impairment through adaptive assistance and task modulation, without requiring additional robotic devices or physical assistance from the therapist during task execution, as reported in another recent study [73]. Our study contributes to offering a more comprehensive view of the user experience by including patient-reported outcomes, therapist evaluations, and cognitive load assessments. This information, together with the evaluation of motor performance carried out with a novel therapeutic approach, will contribute to defining the best practices for the use of this system in rehabilitation protocols that can be tailored to the specific needs of the patient.

## Appendix

Multimedia Appendix 1 Virtual assistance.

## Abbreviations

FMA: Fugl-Meyer Assessment
MRC: Medical Research Council
MT: mirror therapy
NASA-TLX: NASA (National Aeronautics and Space Administration) Task Load Index
sEMG: surface electromyography
USEQ: User Satisfaction Evaluation Questionnaire
VR: virtual reality

## Data Availability

The data that support the findings of this study are available on reasonable request from the corresponding author, DDB.
