# Supplementary figures and images for: Usability, Acceptability, and Feasibility of a Personalized Adaptive Mirror Therapy for Upper-Limb Poststroke Rehabilitation Using Immersive Virtual Reality and Myoelectric Control: Single-Arm Pre-Post Study
Source: JMIR Rehabil Assist Technol. 2026 May 4;13:e81894. doi: 10.2196/81894 (PMC13184599; doi:10.2196/81894)

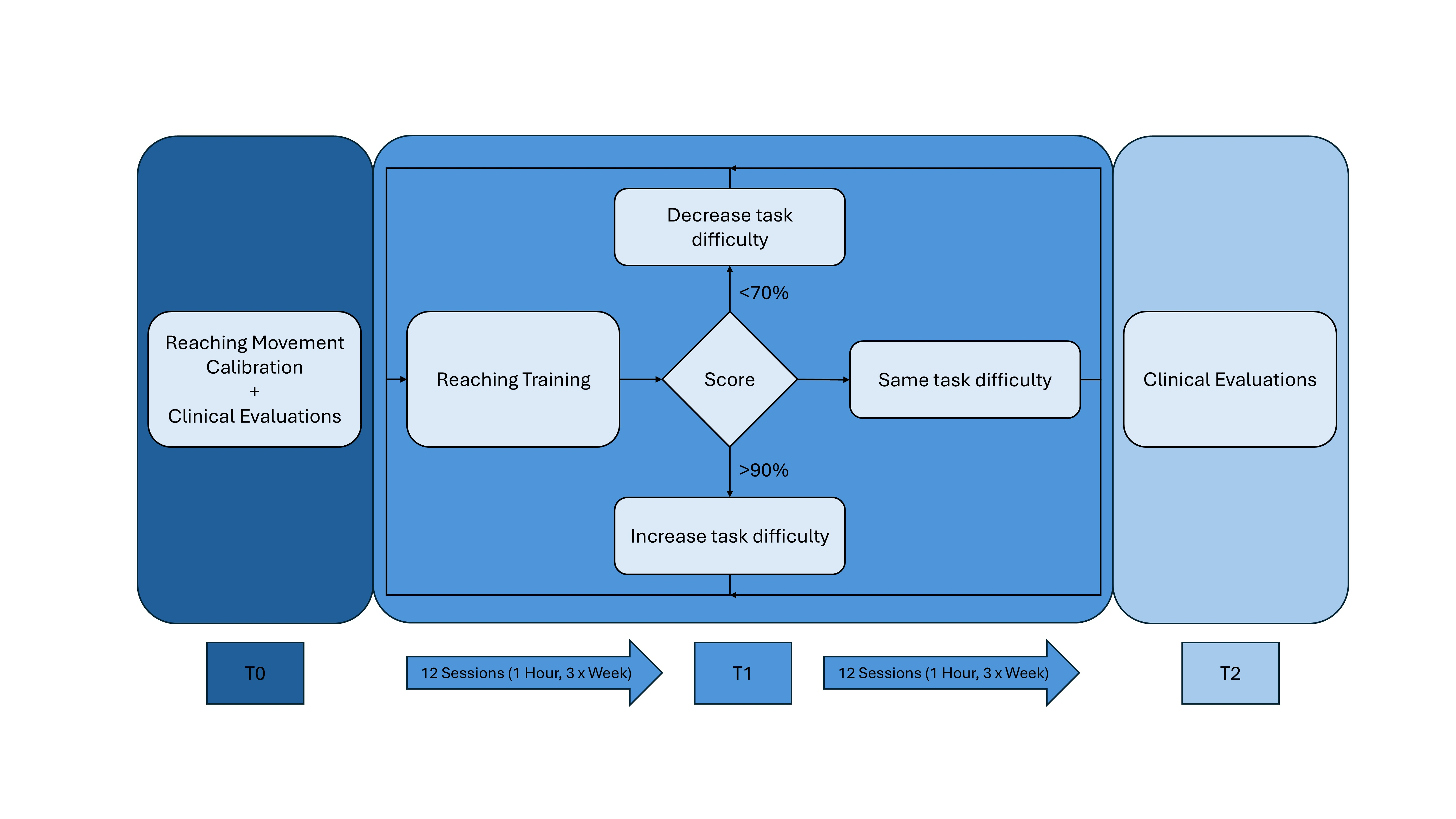

Supplement: Multimedia Appendix 1 [file rehab_v13i1e81894_app1.png]
